# How many holes is too many? A prototype tool for estimating mosquito entry risk into damaged bed nets

**DOI:** 10.1186/s12936-017-1951-4

**Published:** 2017-08-01

**Authors:** James Sutcliffe, Xin Ji, Shaoman Yin

**Affiliations:** 1 Department of Biology, Trent University, Peterborough, ON Canada; 2 Entomology Branch, US Centers for Disease Control and Prevention, Atlanta, GA USA; 3 Department of Mathematics and Statistics, Georgia State University, Atlanta, GA USA; 4 CDC Foundation, Atlanta, GA USA

**Keywords:** Bed nets, Bed net damage, ITNs, Bed net entry risk, Mosquitoes, Mosquito behaviour

## Abstract

**Background:**

Insecticide-treated bed nets (ITNs) have played an integral role in malaria reduction but how insecticide depletion and accumulating physical damage affect ITN performance is poorly understood. More accurate methods are needed to assess damage to bed nets so that they can be designed, deployed and replaced optimally.

**Methods:**

Video recordings of female *Anopheles gambiae* in near approach (1–½ cm) to occupied untreated rectangular bed nets in a laboratory study were used to quantify the amount of mosquito activity (appearances over time) around different parts of the net, the per-appearance probability of a mosquito coming close to holes of different sizes (hole encounter) and the per-encounter probability of mosquitoes passing through holes of different sizes (hole passage).

**Results:**

Appearance frequency on different parts of the net reflected previously reported patterns: the area of the net under greatest mosquito pressure was the roof, followed by the bottom 30 cm of the sides, followed by the 30 cm area immediately above this, followed by the upper two-thirds of the sides. The ratio of activity in these areas was (respectively) 250:33:5:1. Per-appearance probability of hole encounter on all parts of the net was strongly predicted by a factor combining hole perimeter and area. Per-encounter probability of hole passage, in turn, was strongly predicted by hole width. For a given width, there was a 20% greater risk of passage through holes on the roof than holes on the sides.

**Discussion:**

Appearance, encounter and passage predictors correspond to various mosquito behaviours that have previously been described and are combined into a prototype mosquito entry risk tool that predicts mosquito entry rates for nets with various amounts of damage. Scenarios that use the entry risk tool to test the recommendations of the WHOPES proportionate hole index (pHI) suggest that the pHI hole size categories and failure to account for hole location likely sometimes lead to incorrect conclusions about net serviceability that could be avoided by using an entry risk tool of the form presented here instead. Practical methods of collecting hole position, shape and size information for bed net assessments using the tool in the field are discussed and include using image analysis and on-line geometric analysis tools.

**Electronic supplementary material:**

The online version of this article (doi:10.1186/s12936-017-1951-4) contains supplementary material, which is available to authorized users.

## Background

The hundreds of millions of insecticide-treated bed nets (ITNs) distributed in sub-Saharan Africa and other malaria endemic areas are acknowledged to have played a major role in the global malaria control and elimination effort which has reduced the total malaria burden by more than half since 2000 [1].

ITNs work, in part, by providing a physical barrier that prevents host-seeking mosquitoes from gaining access to people inside and infecting them (or being infected by them) while they sleep. The physical protection ITNs provide is complemented by insecticides in the netting that kill, knock down or deter mosquitoes that contact the netting [2]. As ITNs age, they accumulate physical damage in the form of rips and tears [3, 4] and the insecticides with which they are treated are gradually depleted by natural processes and washing [5, 6]. The World Health Organization (WHO) in its 2016–2030 malaria strategy technical paper [7] states, “National malaria programmes need to ensure that all people living in areas where the risk of malaria is high are protected through the provision, use and timely replacement of long-lasting insecticidal nets…”. Unfortunately, the timely replacement of nets is problematic because the impact of physical damage alone or in combination with insecticide depletion on net protectiveness is not well understood. One reason that previous attempts to find predictors of damaged bed net protective value [8, 9] have not been completely successful is our poor understanding of how mosquitoes behave around bed nets and damaged areas in them. Recently, several studies on the behaviour of mosquitoes around bed nets have started to fill in this picture. Using human volunteers and, respectively, a sticky rectangular net and sticky screen squares on an untreated rectangular net, Lynd and McCall [10] and Sutcliffe and Yin [11] showed that the bed net roof comes under greatest mosquito pressure from *An. gambiae*, the lower third of the net sides (vertical surfaces including the ends) come under intermediate pressure and the upper two-thirds of the nets receive the least pressure. Sutcliffe and Yin [11] called these zones of mosquito pressure on the bed net ‘Functional Areas’ (FAs). The ratio of mosquito pressure measured this was for FAs 1, 2 and 3 was approximately 40:10:1. This has implications for how bed net damage should be assessed when deciding on the need for replacement since it predicts that damage on the net roof and lower sides will represent a greater mosquito entry risk than damage on parts of the net that are less frequently visited by mosquitoes, such as the upper sides and ends. Subsequent studies [12, 13] using a three-dimensional mosquito flight tracking system confirm the strong focus for host seeking mosquitoes on the net roof and provide improved resolution of the behaviours that bring mosquitoes into contact with the net. These include ‘bouncing’ flight, a behaviour consisting of rapid close contact flight across the net roof where the mosquito appears to bounce and skim over the surface, and ‘visiting’ flights which may start at some distance from the net but that result in contact at a higher angle than bouncing flight.

Knowing where on the net mosquitoes go and how they get there is important for purposes of assessing entry risk but it is also important to know how likely they are to enter the net through holes they encounter. Sutcliffe and Colborn [14] provided some insights into this using video recordings to study how *An. gambiae* females interact with holes of different sizes on vertically and horizontally-oriented netting in small scale behavioural arenas set up around a human experimenter to simulate the sides and roof a bed net. They showed that as diameter of holes on the net sides and, to a lesser extent, holes on the roof, get smaller, they become less passable for the mosquito than the reduced area alone can account for. They suggested that mosquitoes flying around net holes sometimes contact the hole edges with their bodies, wings or appendages and this may cause them to be deflected away. They hypothesized a hole ‘edge effect’ that makes such disruptive in-flight collisions more likely as the hole size decreases (and as the ratio of hole edge to hole area increases). This suggests that holes in netting may rapidly become more passable as their width or diameter increases.

Sutcliffe and Colborn [14] also showed that the heat and humidity plume from the experimenter inside the simulated net (and, presumably, by extension, from the occupants of real nets) rose strongly upward in still air apparently accounting for the greater activity of mosquitoes in the overhead arenas than in the arenas to the side and for the greater ability of mosquitoes on the roof to pass through small holes despite the edge effect. This too has implications for net entry risk assessment because it suggests that the mosquito distribution patterns and hole passage rates on different parts of the net may be influenced by air movement around the net.

Taken together, these observations suggest a framework within which the problem of assessing damaged net entry risk can be approached since they show that a mosquito must go through specific steps to enter a damaged bed net: (1) it must come close to the net, (2) it must come close to a hole in the net and (3) it must pass through the hole. If the factors that make each of these steps more or less likely can be identified and quantified it should be possible to construct models to predict mosquito entry risk for bed nets in a wide range of physical conditions. For present purposes the steps listed above are referred to in order as (1) net ‘appearance’, (2) hole ‘encounter’ and (3) hole ‘passage’. In this study, video recordings of near-net behaviour were used to determine reliable quantitative predictors (aspects of net location, hole size, hole shape, etc.) of these events for female *An. gambiae*. The strongest of these predictors are then incorporated into a prototype net entry risk assessment tool.

## Methods

### Experimental insects

Mosquitoes were drawn from stock colonies of *Anopheles gambiae* s.s. (G3 strain) maintained by the Malaria Branch at the Centers for Disease Control and Prevention (CDC) in Atlanta, Georgia. Colonized larvae, pupae and adults were reared at 28 °C on a 12 h:12 h light:dark cycle with a 30 min artificial sunrise and sunset. Colony adults emerged directly into 4 L cylindrical cardboard containers and were provided with carbohydrates ad libitum in the form of 10% corn syrup in water.

### Experimental setting and recording system

**Fig. 1:**
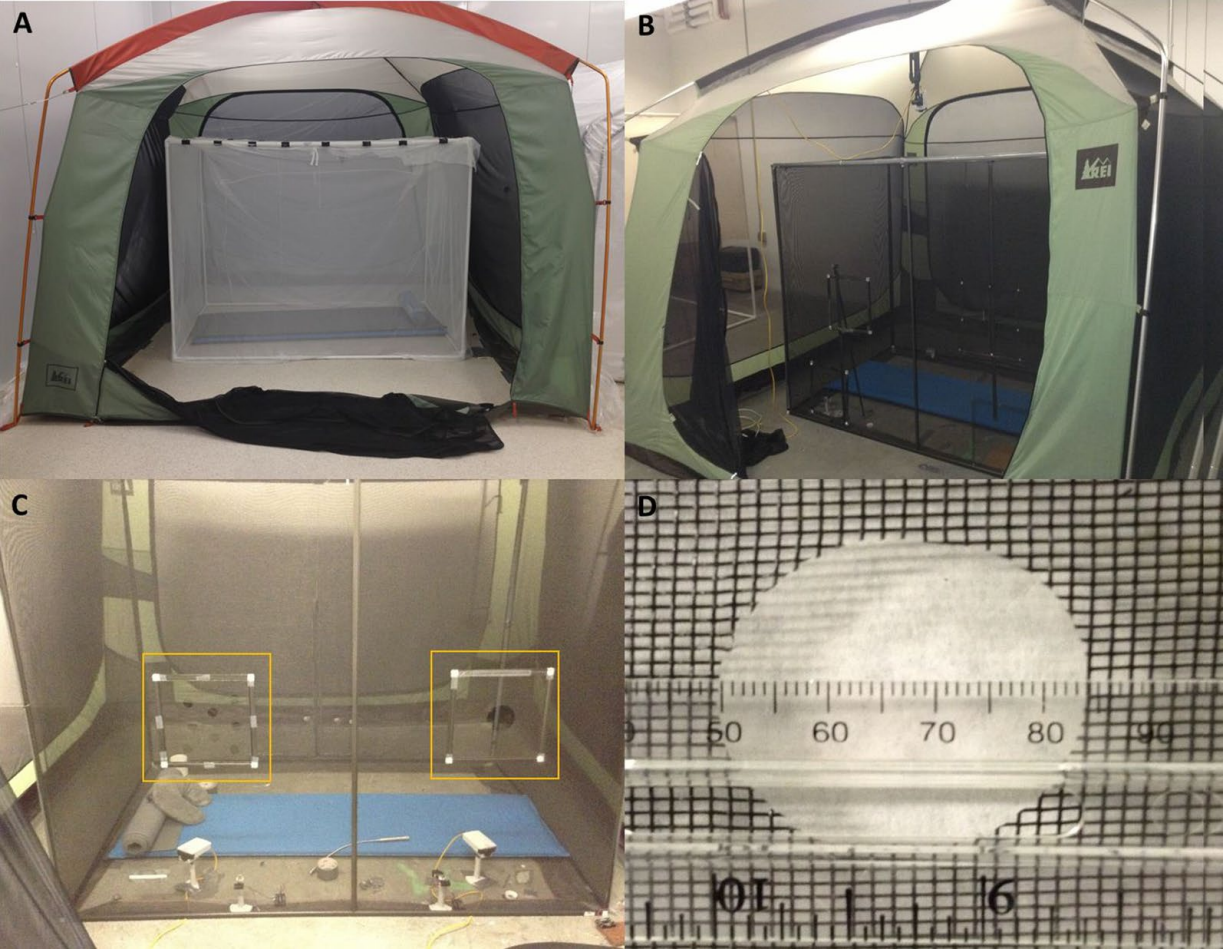
Bed nets in mosquito-tight tents. **a** Tent housing a 150 cm × 180 cm × 130 cm polyester net on PVC support frame. **b** Tent housing fibreglass net of the same dimensions as the polyester net. **c** Front side of fibreglass net. Frame modules (in *yellow boxes*) at two locations fitted, in this instance, with two sizes of round holes. Air mattress, fibre optics light, tape etc. for net occupant. Two cameras located on the floor inside the net for illustration. **d** Close-up of one 35 mm diameter circular hole in fibreglass mesh

All three parts of the study were based on video recordings of mosquito behaviour around a bed net erected in a 3 m × 3 m × 2.1 m tall REI^®^ screen house (‘tent’) (REI catalog #794-289-0018) (Fig. 1a, b) in a large open experimental room (approx. 10 m × 5 m × 5 m high) operating at ambient building conditions (approx. 23–26 °C, 40–65% relative humidity). Air movement in the open room was slight and further minimized inside the tent in which all recordings were conducted.

Recordings for objective 1 (frequency of appearance on the net) used an untreated polyester net (150 cm high × 180 cm long × 130 cm wide) mounted on a frame made of PVC tubing which was used to keep the top and sides taut and flat (Fig. 1a). Objectives 2 and 3 (frequency of hole encounter and frequency of hole passage, respectively) used a similar size bed net made of (insecticide-free) fibreglass screen panels (grey window screening mounted in aluminum window screen frame material) assembled into a bed net shape (Fig. 1b, c) and featuring holes that had been cut to pre-determined sizes (Fig. 1c, d).

**Fig. 2:**
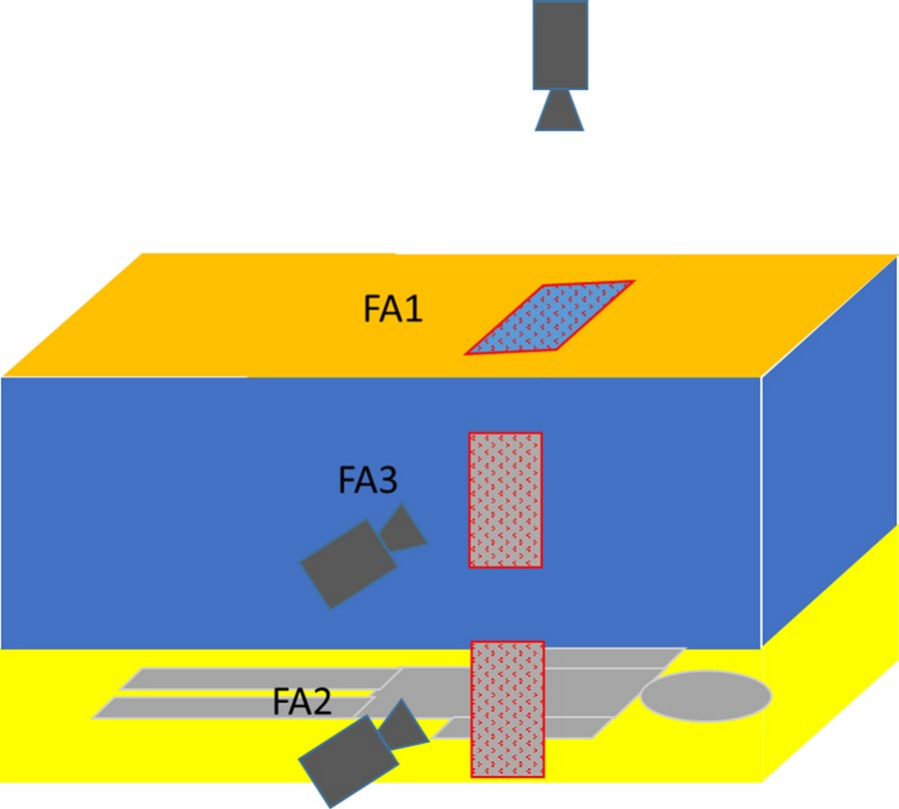
Diagram of an occupied bed net. Net is divided into areas of greatest (FA1), intermediate (FA2) and least (FA3) mosquito pressure. Cameras are directed at red stippled 60 cm × 30 cm video fields in each FA

Video recordings were made with Noldus Media Recorder^®^ (version 2.1) software and used up to four Axis IP cameras (Axis Corporation, Sweden, Model M1144-L) aimed at selected areas of the net (Fig. 2). Recordings were made with room lights off and, with the exception of the laser illumination system (see below), no other sources of illumination.

**Fig. 3:**
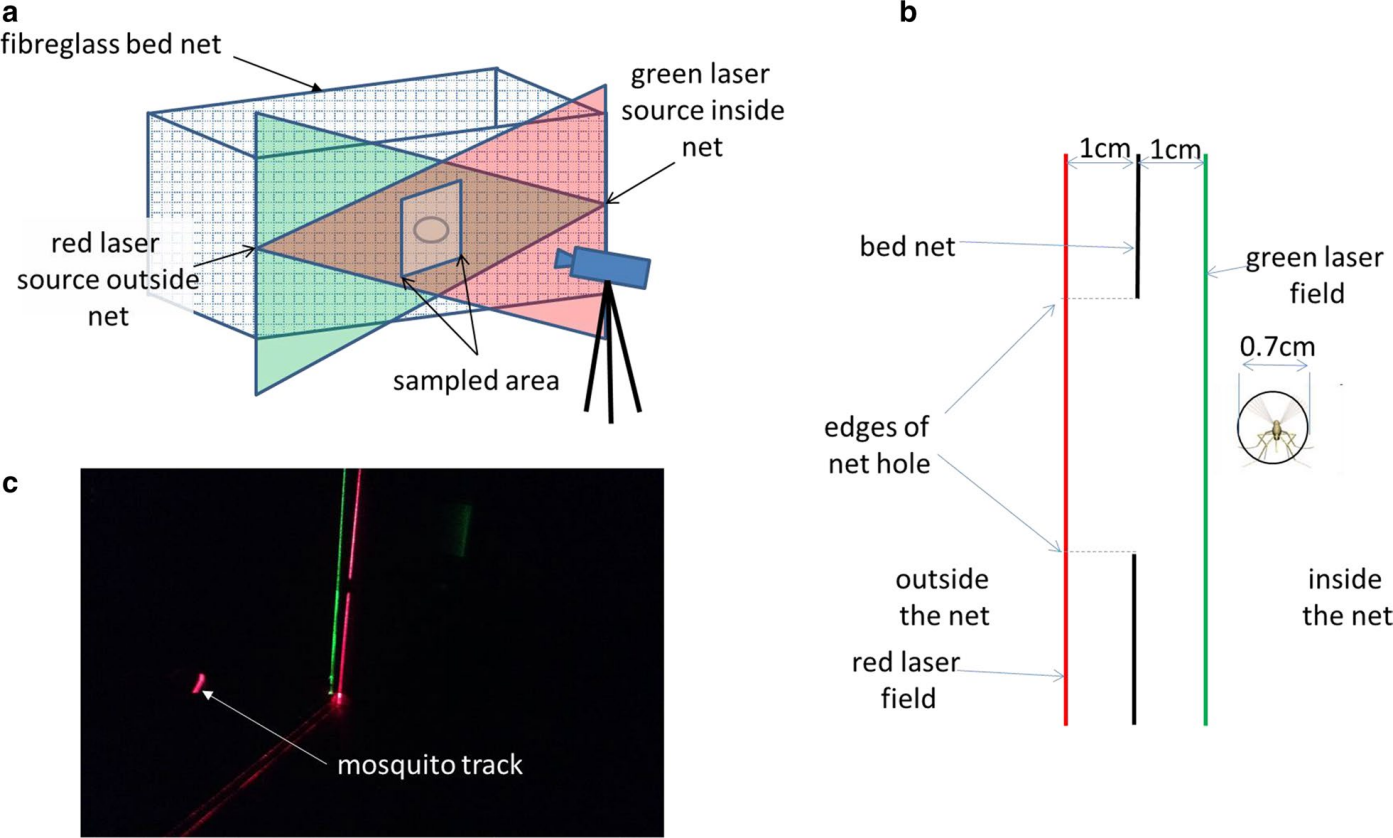
Arrangement and operation of laser illumination system. **a** Diagram of bed net depicting laser sources directed across the outside (*red*) and inside (*green*) of the net side and a camera trained on the sampled area around a hole in the net. Number and configuration of laser fields, sampled areas and holes varied depending on recording session (see “Methods”). **b** Bed net (*black line*) and a hole in it (gap) viewed end-on and showing arrangement and approximate spacings of red laser field (outside of the net) and green laser field (inside of the net). Approximate *An. gambiae* flight profile [14] shown for comparison. **c** Photograph of flying mosquito captured immediately above bed net in red laser field. Reflected *red* and *green laser lines* visible on floor and vertical edge of corner of bed net

**Fig. 4:**
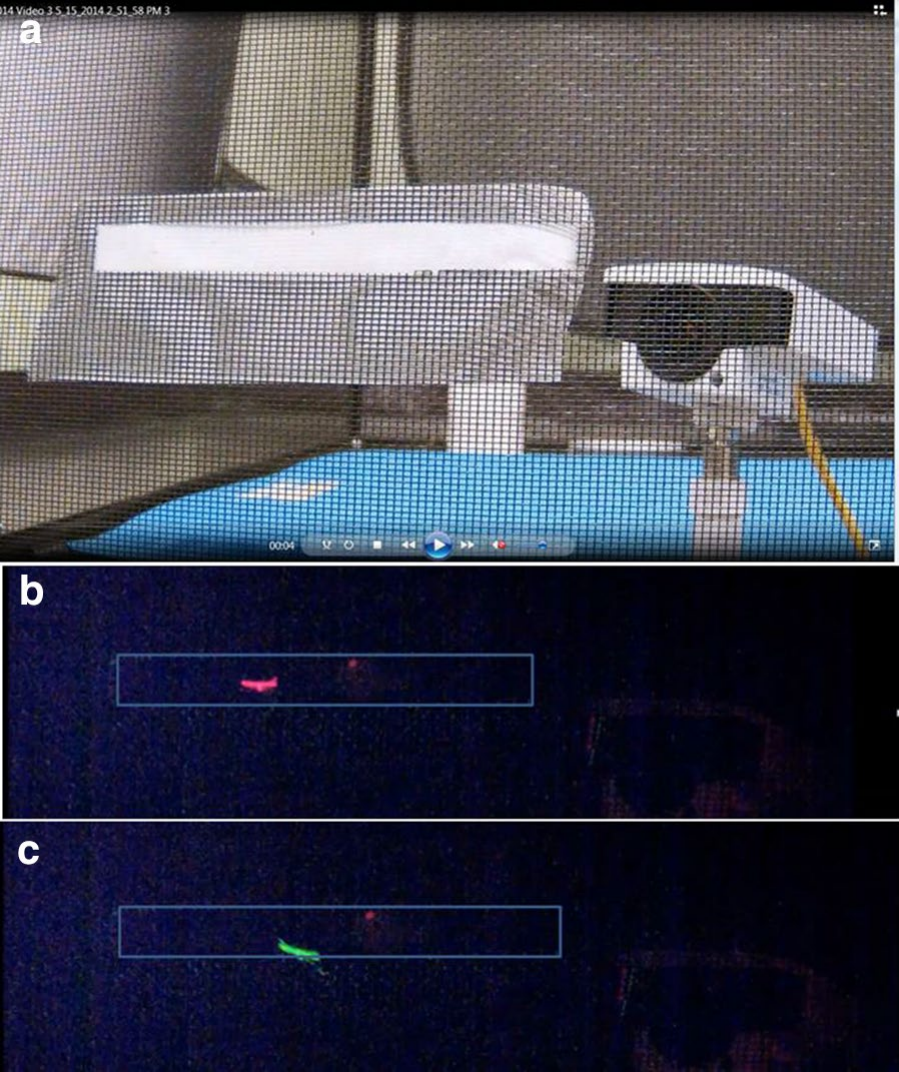
Video capture of mosquito encountering hole and passing through. **a** 12 by 120 mm rectangular hole in lower third of fiberglass net from video taken by a camera outside the net. A camera inside the net (placed for purposes of illustration), part of a blue air mattress and the back of the tent are also visible. Hole is temporarily covered with tape to prevent premature mosquito entry. **b** Mosquito on outside of net captured in *red laser* field as it encounters the hole (outlined). **c** Mosquito in **b** a few frames later as it is captured by *green laser field* inside the net indicating hole passage

The laser illumination system consisted of 3V red line lasers (Apinex, 5 mW, 650 nm, 90^o^ fan angle) directed along the outside of the net within 1.0–1.5 cm of its surface (Fig. 3a, b). These permitted detection of mosquitoes that were flying close to net surfaces (Fig. 3b, c). For objective 3 (hole passage) recordings, in addition to the red lasers on the outside of the net, a 3V green line laser (Apinex, 5mW, 532 nm, 90^o^ fan angle) was directed across each hole on the inside of the net approximately 1.0–1.5 cm from its inner surface. These allowed detection of mosquitoes as they passed through holes (Fig. 4).

### Recording session procedures

Recording sessions for all objectives were run in the late afternoon using 4–8 day-old nulliparous female mosquitoes that had not previously been blood-fed. In the morning of the recording day, a container of several hundred mosquitoes was taken from the general colony and held with access to water only.

Approximately 30 min before the start of recordings, groups of up to 50 female mosquitoes were gently mouth-aspirated with a HEPA-filtered aspirator from their container into several screw-top polystyrene vials (approximately 3 cm wide × 8 cm tall). To help ensure they were blood hungry, females were drawn from those that landed on, and tried to probe through, the containment cage’s mesh sleeve draped across a bowl of warm water. Vials, with lids loosened, were placed on the floor of the tent housing the bed net on the side away from the tent entrance and from the cameras recording activity on the net sides.

After lasers had been turned on and their alignments checked, the recording system was started, the room lights were turned off and the experimenter, wearing shorts, a short-sleeved shirt and socks (no shoes) entered the tent, closed it and entered the net. The experimenter then released the mosquitoes by reaching out from inside the net with a short stick and tipping the vials over. After securing the net, the experimenter laid on a cot or, in the case of hole passage sessions, on an air mattress inside a small self-supporting untreated net, inside the main net. The experimenter faced upward and remained as still as reasonably possible throughout the recording period (60–90 min). The experimenter then exited the net and the tent and stopped the recording system.

#### Objective 1—mosquito appearance frequency on the net

To get measurements of activity levels on the net, simultaneous recordings were made with separate cameras directed at areas within each FA; specifically, at the roof above the net occupant’s torso (FA1), at the bottom third of the net side at mid-torso level (FA2) and at an area in the upper two-thirds of the net side also at mid-torso level (FA3) (Fig. 2). Each camera’s field took in two contiguous 30 cm × 30 cm sampling areas plus a small surrounding area.

#### Objective 2—determinants of hole encounter frequency

Hole encounter frequency was measured on the fibreglass net using the recordings that were also used to measure hole passage frequency (“Objective 3”, see below). In these recordings, to allow resolution of hole encounters and passages, the camera’s field of view included the hole and an area of two to four centimetres around it; accordingly, actual areas recorded ranged from 36 to 445 cm^2^. To be included in the hole encounter analysis, recordings (or sections of recordings) had to be free of background reflections (primarily from net occupant’s movements) that might have been confused for mosquito activity by the automated detection system (see below). In addition, overall mosquito activity levels in the videoed areas had to be high enough to yield the equivalent of at least 500 mosquito appearances in the extended 30 cm × 30 cm square centered on each hole in question.

#### Objective 3—determinants of hole passage

Test holes on the side of the fibreglass net were placed in locations in FA2 which Sutcliffe and Yin [11] showed are in the area where most *An. gambiae* activity on the net sides is concentrated. Holes in the roof were placed roughly in the middle of the roof (FA1).

Holes of pre-determined size and shape were either cut directly into the fibreglass of the net or in pieces of fibreglass screen mounted in 30 cm × 30 cm framed modules that could be placed into and removed from the net as needed (Fig. 1c, d). Fibreglass was chosen for this purpose because its resistance to distortion and stretching (compared to polyester and polyethylene netting of commercial nets) preserved desired hole size and shape over several recording sessions (Fig. 1d). The fibreglass net was also more practical for this objective since the many hole size, shape and position combinations needed would have consumed more untreated fabric nets than were readily available. Mosquito activity patterns around the fibreglass net were similar to those observed around the polyester net and mosquito responses to the edges of holes in the fibreglass netting were similar to those reported around similar sized holes in polyester netting [14].

Rectangular holes were the most extensively studied shape for this objective. Rectangles were used because they are regular shapes that are easily cut into the mesh and their dimensions can be varied so that a range of lengths, widths, perimeters and areas can be represented. Hole length and width (‘width’ used herein for the lesser dimension) increments were determined by the fibreglass mesh which consisted of 1.4 by 1.9 mm rectangular holes (including the fibreglass strand).

Round and triangular hole dimensions were chosen based on preliminary models of hole passage derived from experiments with rectangular holes. Triangular holes were isosceles in shape. The target number for encounters for any given hole size-shape-location combination was 50 or more collected over at least two recording sessions.

### Analysis of videos

Videos from all sessions were analysed using Noldus Ethovision^®^ (ver. 10.1) motion tracking software. Ethovision detection settings and thresholds were chosen based on preliminary trials. In all cases detection used the ‘Differencing’ mode, the subject was set to be ‘brighter’ than the background, sensitivity was set at 30 and the minimum subject size was 30 pixels. The sections of recordings that were analysed excluded periods at the beginning and end of sessions when the experimenter was moving in view of the cameras and totalled 45–70 min depending on the session.

After preliminary analysis with Ethovision was complete for each recording, the result was examined by eye for artifacts; in particular, for false detections caused by experimenter movement in the net. Where these totalled to no more than 30 s out of the entire recording, they were edited out of the record before proceeding. Where they exceeded 30 s, the recording was not used.

For objective 1 and 2 analyses, mosquito activity was measured as the number of times at least one mosquito appeared in the red laser field in the sampled area in a 1/30 s video frame. This count was then adjusted by the proportion of ‘missed samples’ reported by the Ethovision system (up to 75%) and for an equivalent release of 200 mosquitoes. The result is expressed as mosquito ‘appearances’. For objective 1 analyses, the sampling areas were adjacent 30 cm × 30 cm squares set up as Ethovision ‘zones’. For objective 2 analyses, where sampled areas varied in size (36–445 cm^2^), mosquito activity measured as above was adjusted to the equivalent number of appearances for the 30 cm × 30 cm field of the net surrounding the hole. This made it possible to compare activity levels directly from different parts of the net to one another and to activity measures from objective 1 measurements and it made it possible to estimate hole encounter on a per-appearance basis.

For hole passage analyses (objective 3), each test hole was defined as an Ethovision ‘zone’. If a mosquito, or any part of it, impinged on the zone in the laser field it was considered to have ‘encountered’ the hole. If a mosquito encountered a hole, moved away from it and then returned to impinge on it again, a separate encounter was scored. Because many mosquitoes were released at the same time, it was not possible to ascribe specific encounters to particular mosquitoes; thus, each encounter is interpreted as an independent event.

If, after an encounter, a mosquito appeared in the green laser field inside the net within a few frames, it was considered to have passed through the hole and entered the net (i.e. hole ‘passage’ had occurred). In a few instances, mosquitoes were seen first in the green laser inside the hole and then in the red laser. These were considered to be passages from inside the net to the outside (i.e. net ‘exits’).

All Ethovision-detected encounters and passages were confirmed by direct visual inspection of the video recordings because various sources of noise (particularly dust particles in the green laser) were sometimes interpreted as mosquitoes by the Ethovision system.

### Data analysis

Both dependent variables (hole encounter per appearance and hole passage per encounter) were modeled against a number of hole parameters including width, length, area and various combinations of these parameters using linear regression.

## Results

### General

Mosquito activity level in the tents varied greatly from session to session but relative mosquito pressure on the net consistently conformed to previously reported patterns [11]. Casual observations revealed that mosquitoes were largely inactive in the tent unless someone occupied the bed net.

### Objective 1

**Fig. 5:**
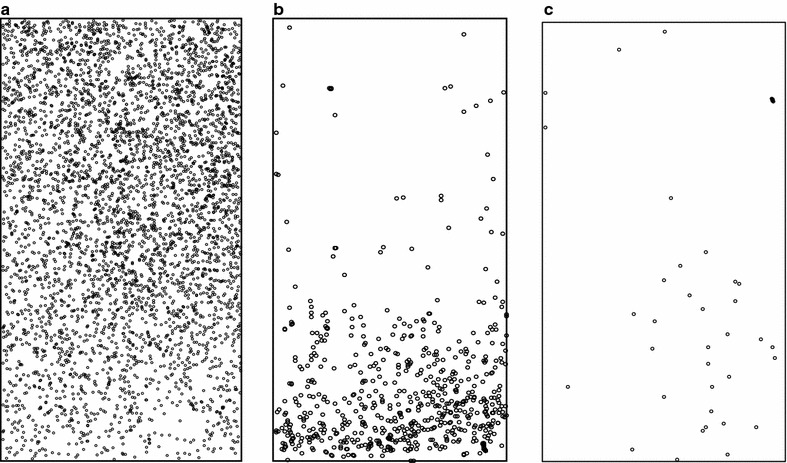
Accumulated Ethovision mosquito detections over 64 min in concurrent ‘appearance frequency’ (Objective 1) recordings on **a** the roof (FA1), **b** the lower side (FA2) and **c** the upper side of occupied polyester bed net. All fields measure approximately 30 by 60 cm on the net. Lower halves of rectangles in **b** and **c** are closer to the floor

**Fig. 6:**
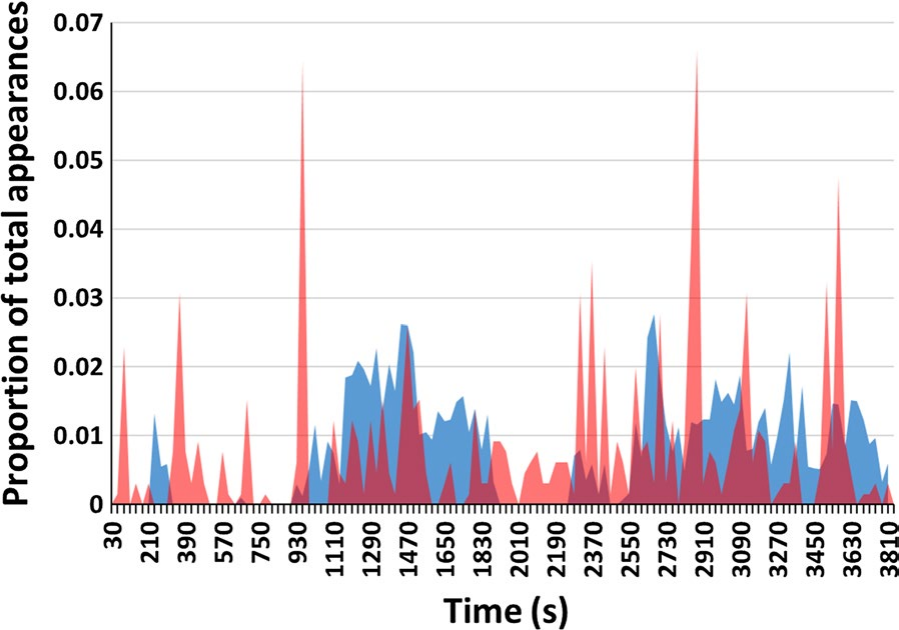
Proportion of total appearances in successive 30 s periods of the session illustrated in Fig. 5. Total activity for roof location (FA1) (*blue*) = 13,294 appearances and for low side location (FA2) (*red*) = 1264 appearances. FA3 appearances were not plotted because their very low total would result in distorted proportions

While absolute mosquito activity levels varied from session to session, Shapiro-Wilks tests of normality confirmed that appearances at all locations were normally-distributed. In all 13 sessions the roof location had by far the most activity averaging 14,450 appearances per hour (median 12,290; range 4928–35,097). This was 13.2 times more appearances than the average for the low side location (FA2) (mean 1090; median 1244, range 70–2400) and 253.5 times more than for the upper two-thirds side location (FA3) (mean 57; median 61; range 16–114) (see Fig. 5 for examples). For all locations, activity in the sampled areas fluctuated independently over the course of each session (see Fig. 6 for an example). Fluctuating activity may reflect the entry of individual mosquitoes into the host seeking mode (and the departure from it by others) or it may be the result of shifts of mosquito activity to areas of the net not under observation. Such shifts could have been due to subtle changes in the odour plume rising from the net occupant. The number of mosquitoes that contributed to recorded activity is not known; however, because during hole entry sessions up to 30% of released mosquitoes entered the net in the course of an hour, it follows that well more than 30% of released mosquitoes contributed to the recorded activity at some point or points throughout sessions.

Closer examination of FA2 revealed that, in all but one of 13 cases, activity was concentrated on the lower of the two 30 cm × 30 cm areas (Fig. 5) (the mean ratio of lower half to upper half activity = 6.5; median 6.3; range 0.5–15.1; both normally distributed).

### Objective 2

As was the case for the polyester net used in objective 1 recordings, mosquito activity level varied from session to session on the fibreglass net but in all cases was much higher at the roof location than on the sides. In the 93 recording sessions, appearances in the 30 cm × 30 cm square around each hole ranged from as few as 100/h at side locations to almost 30,000/h at the roof location (reflecting the pattern seen in objective 1 activity measurements).

In the 114 sections of recordings that were of sufficient quality and in which equivalent appearances in the 30 cm × 30 cm square around holes totalled 500 or more, values for encounters per appearance were tested against hole area, hole perimeter, hole length and hole width. The location of the hole did not have a significant effect for any of these; consequently, data for the roof and sides were pooled in analyses. Encounters per appearance increased significantly with increases in all parameters (Table 1) with hole area and hole perimeter having the strongest relationships.

**Table 1:** Summary of linear regression analyses for per appearance rectangular hole encounter probability (y) on roof and sides combined

Model	Coefficient (b)	*p* value	R^2^
y = a + b × length	3.05E−04	<0.001	0.644
y = a + b × width	9.99E−04	<0.001	0.300
y = a + b × perimeter	1.46E−04	<0.001	0.746
y = a + b × area	8.99E−06	<0.001	0.857

### Objective 3

Hole passage data were collected from analyses of sections of videos ranging from 15 to 70 min (depending on video quality) of 162 recordings made in the 93 recording sessions. Rectangular test holes were concentrated in the smaller sizes because hole passage rates changed most rapidly in this range. While the target minimum for encounters for each hole size-location combination was 50, actual encounters for some were considerably larger or, in some cases, smaller, than this (Table 2) for several reasons. Achieving minimum targets (and overshooting them) happened more quickly on the net roof where mosquito activity was greatest. Activity on the sides, on the other hand, was relatively low; thus, more time and recording sessions were required, especially for smaller holes, for the minimum 50 encounters to be accumulated. Target numbers for some hole sizes on the net sides were not achieved for this reason. In other cases, fewer than 50 encounters were achieved because actual hole dimensions differed from intended dimensions due to cutting errors that were only discovered after recordings had been completed.

**Table 2:** Summary of hole passage per encounter experiment outcomes by hole dimension for rectangular holes in the net sides and roof and circular and triangular holes in the net side

Width (mm)	Length (mm)	# encounters	# passages	Prop’n passages
Sides, rectangular				
6	8	76	0	0.00
	10	53	0	0.00
	25	131	2	0.02
	210	100	2	0.02
11	11	112	10	0.09
15	15	26	6	0.23
	85	120	41	0.34
	120	53	20	0.38
	210	24	8	0.33
17	19	43	12	0.28
20	32	87	34	0.43
	40	71	34	0.48
	113	56	28	0.50
35	84	102	56	0.55
	152	81	49	0.60
	210	77	49	0.64
60	82	70	45	0.64
	120	69	50	0.72
	210	13	8	0.62
Roof, rectangular				
4	6	68	0	0.00
6	6	147	0	0.00
	8	314	9	0.03
	13	165	7	0.04
	21	90	9	0.10
	25	170	17	0.10
	40	53	5	0.09
	60	56	4	0.07
	77	140	9	0.06
	148	63	4	0.06
8	13	234	21	0.09
	38	30	2	0.07
	60	45	9	0.20
11	13	202	35	0.17
	38	31	4	0.13
	59	83	38	0.46
	80	46	16	0.35
13	17	136	62	0.46
	40	72	27	0.38
	160	118	45	0.38
15	17	117	29	0.25
	40	63	18	0.29
	60	69	32	0.46
	76	55	22	0.40
	160	52	23	0.44
21	22	103	48	0.47
	40	77	36	0.47
	60	66	29	0.44
	77	61	33	0.54
	160	95	39	0.41
30	30	70	49	0.70
	60	94	51	0.54
	80	37	26	0.70

Base (mm)	Av. width (mm)	# encounters	# passages	Prop’n passages
Side, triangular, 210 mm				
25	15	68	19	0.28
36	21	42	17	0.41
80	42	90	49	0.54

Diameter (mm)	Length (mm)	# encounters	# passages	Prop’n passages
Side, round				
8	NA	43	2	0.04
35	NA	120	61	0.51
40	NA	153	90	0.59
70	NA	43	31	0.72

Maximum roof hole size tested was smaller than for the sides (Table 2) because holes larger than this on the roof would have resulted in an unacceptably large proportion of the released mosquitoes getting inside the net within a few minutes thus depleting the remaining cohort outside and further slowing the accumulation of encounters at side holes.

The per-encounter probability of passage through rectangular holes was tested against several hole parameters (hole width, hole perimeter, hole length, hole area, etc.—Table 3). Of these, linear regression analyses showed that hole width had the strongest relationship to hole passage rate for both side holes (R^2^ = 0.72, z = 7.2, p < 0.001) and roof holes (R^2^ = 0.81, z = 13.9, p < 0.001). Confidence limits for the coefficients of increase for side holes (0.011) and roof holes (0.025) were non-overlapping (0.008 – 0.014 vs. 0.022 – 0.029, resp.) indicating that per-encounter probability of passage for any given width was significantly greater for roof holes. In addition to rectangular holes, several sizes of circular holes and triangular holes were tested in the net side (Table 2).

**Table 3:** Summary of linear regression analyses for per encounter hole passage probability (y) for rectangular holes on the roof and for rectangular, round and triangular holes on the sides

Model	Coefficient (b)	p value	R^2^
Roof, rectangular holes			
y = a + b × length	1.445E−03	0.07856	0.0673
y = a + b × width	2.514E−02	<0.001	0.8122
y = a + b × ln (width)	3.434E−01	<0.001	0.8621
y = a + b × perimeter	9.759E−04	0.0103	0.1678
y = a + b × area	1.643E−04	<0.001	0.3920
Side, rectangular holes			
y = a + b × length	1.479E−03	0.0476	0.1650
y = a + b × width	1.134E−02	<0.001	0.7187
y = a + b × ln (width)	3.020E−01	<0.001	0.9256
y = a + b × perimeter	8.557E−04	0.00705	0.3176
y = a + b × area	5.312E−05	<0.001	0.5075
Side, triangular holes			
y = a + b × mean width	0.009	0.21	0.89
y = a + b × ln (mean width)	2.430E−01	0.14	0.95
Side, round holes			
y = a + b × diameter	3.192E−01	0.0025	0.99
y = a + b × ln (diameter)	3.192E−01	0.0025	0.99

Instances of mosquitoes appearing first in the green laser and then, within a few frames, in the red laser were interpreted as exits by mosquitoes that had entered earlier. Exits were strongly positively correlated with hole width and occurred virtually exclusively through holes on the lower sides (FA2) where they were approximately 10% as frequent as entries compared to less than 1% as frequent on the roof where only two exits were observed. In some cases, mosquitoes that exited the net were observed to re-enter.

## Discussion

### Objective 1—activity levels

Using sticky squares to sample *An. gambiae* females coming to different parts of occupied bed nets, Sutcliffe and Yin [11] resolved three functional areas (FAs) in terms of mosquito pressure. Expressed in terms of expected average mosquito catch per hour on 30 cm × 30 cm sticky squares placed in each FA, pressures were 29.5 for FA1 (middle section of roof), 6.9 for FA2 (lower third of net plus head and foot ends of the roof) and 0.7 for FA3 (upper two-thirds of the net) yielding a ratio of activity of approximately 1:10:40 for FA3:FA2:FA1, respectively. While this illustrates that different parts of the net come under dramatically different mosquito pressures, these results could not be converted directly to quantitative FA-specific measures of mosquito activity for use in a quantitative tool. This is because the sticky square sampling method continually removes mosquitoes from the cohort likely leading to underestimates of activity in the most frequented areas. The present study bridges this gap providing direct quantitative measurements of mosquito activity in each FA. The ratio of FA3:FA2:FA1 activity is approximately 1:20:250 confirming that the sticky square method underestimated activity in areas with the greater pressure. Despite this, the sticky square method is simple and could be used in the field to map mosquito pressure for other important vector species if a conversion factor between sticky square catch and appearances was available. The data presented here, while not extensive, suggests a conversion rate of about 500 appearances to 1 mosquito caught on a sticky square.

As noted above, these results provide activity measurements for each FA which can be taken forward into the net entry risk model. They also make it possible to ‘fine-tune’ the map of *An. gambiae* pressure on the net because they show that pressure varies significantly within the lower third of the net (FA2) where it is about 6.5 times greater on the lower half (0–30 cm height) than the upper half (30–60 cm height). Accordingly, FA2 can be sub-divided into ‘FA2-low’ and ‘FA2-high’. The FA3:FA2-high:FA2-low:FA1 per unit area activity ratios can be re-calculated as approximately 1:5:33:250. This yields the following hourly activity values (for groups of 200 mosquitoes) which were used to develop the model: FA1 = 14,450, FA2 low = 1900, FA2 high = 300 and FA3 = 60.

### Objective 2—hole encounter

**Fig. 7:**
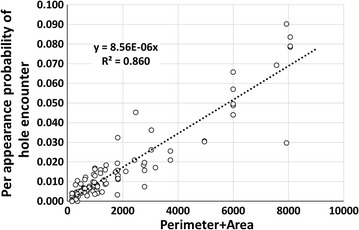
Per appearance probability of encounter with rectangular holes on net side and roof plotted against hole perimeter + area values

Hole encounter was almost equally well-predicted by hole perimeter and hole area (Table 1). Perimeter as a predictor may apply for mosquitoes flying along the net surface as they do in ‘bouncing’ flight. Hole area as a predictor, on the other hand, may apply better to mosquitoes contacting the net (or the plane of the net) in ‘visiting’ flights from above or from the sides (see Parker et al. [13] for descriptions of these flight modes). Since both behaviours occur, both predictors appear to have a place in the model. The relationship of hole encounter and the combined perimeter + area predictor, is significant (t = 25.9, p < 0.001) and accounts for 86.0% of the variation in encounters (Fig. 7). This is not a better fit than the area parameter achieves on its own but for the reasons explained above, the combination would seem to be needed to cover as wide a range of hole shapes as possible, including ones not specifically tested here. As a check on the validity of the combined predictors, per-appearance encounter probability for a hypothetical 30 cm × 30 cm hole, which should be 1 since the hole and the sampling area would be the same size, was tested. The extrapolation yields an encounter probability of over 0.8 which, given the variability of mosquito behaviour in general and the extrapolation distance, is reassuringly close.

### Objective 3—hole passage

That width is such a strong predictor of hole passage may be explained by the edge effect hypothesized by Sutcliffe and Colborn [14]. They performed video experiments in small behavioural arenas that showed that passage through holes in screen over the ends of the arenas may have been affected by collisions of mosquitoes with the hole edges and this ‘edge effect’ became more prominent as hole size decreased (and the ratio of hole edge to hole area increased). They proposed that the influence of this effect is greater for vertically-oriented holes (such as those on the net sides) than for horizontally-oriented holes (such as those on the roof) because, for the latter, the effect is partly offset by the fact that mosquitoes drop vertically through roof holes with a ‘gravity assist’ and because the odour-laden plume from the net occupant rises vertically in still air perhaps providing orienting cues to mosquitoes on the roof that are not available to mosquitoes at the net sides. As a result, on average, the per-encounter passage rate is almost 20% greater for roof holes compared to side holes of the same width further adding to the importance of damage to the bed net roof.

While the primary interest of this study was mosquitoes entering the bed net, the significant number leaving it, especially through larger holes near the bottom of the net was unexpected and deserves some attention. Many mosquitoes leaving the net appeared to do so through the same holes near the bottom of the net that they had just entered. Sutcliffe and Colborn [14] showed that a human subject’s heat and moisture plume rises in still air. Given this, mosquito entry through holes in the net sides appears not to be actively mediated by host cues but, rather, to be the consequence of unoriented flight against the net. If so, in these experiments, ‘inside the net’ and ‘outside the net’ would not be qualitatively different for the mosquito and random flight could readily have taken them out again. In contrast, mosquitoes entering through roof holes are surrounded by the rising host odour plume and drop more or less straight down to encounter the occupant or, in this case, the top of the supplementary net over the occupant, where host odour responses may tend to keep them focussed. In addition, normally mosquitoes entering a bed net blood feed and quickly become quiescent. In these experiments, however, mosquitoes were unable to blood feed and may have remained more active as a result. This, in turn, could have resulted in more mosquitoes leaving the net than would normally be the case. Net exit is not built into the model presented here for several reasons—(1) it is not clear how it should be interpreted, —(2) the recording set-up was not designed to monitor activity on the inside of the net and —(3) it seems prudent not to include factors in the tool that might tend to cause it to underestimate net entry risk.

**Fig. 8:**
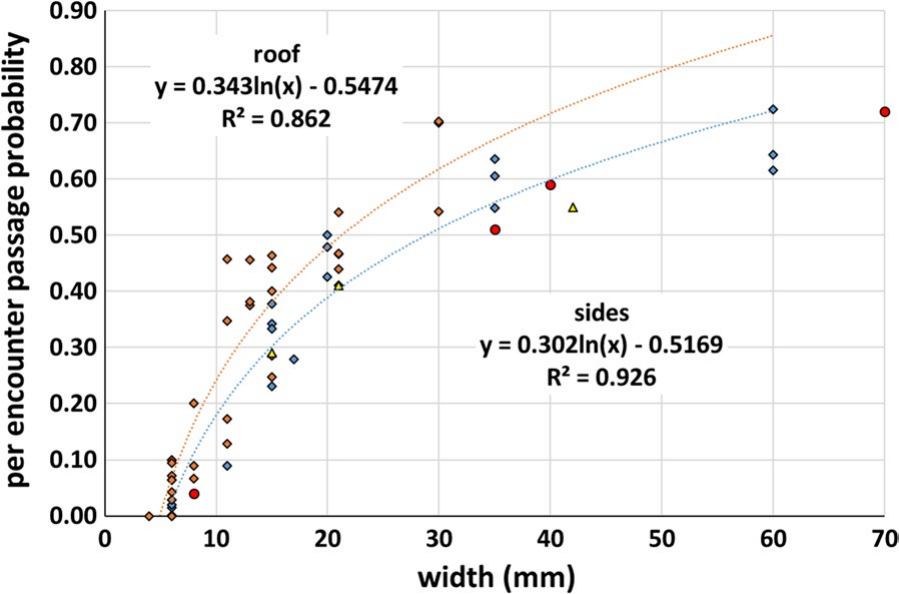
Per encounter probability of hole passage through rectangular holes on the roof (*brown diamonds*) and the sides (*blue diamonds*) plotted against hole width. Per encounter probability of hole passage for circular holes (*red circles*) and triangular holes (*yellow triangles*) plotted against hole diameter and average width, respectively

For modelling purposes, per-encounter passage data for width of rectangular holes were fitted to log-transformed linear models which improved fit to account for 86.2 and 92.6% of the variation for roof and side hole passage, respectively (Table 3; Fig. 8). In these plots, the y-intercepts are 0.52 cm for roof holes and 0.55 cm for side holes reflecting the fact that mosquitoes in this study virtually never passed through holes of this width. This supports the WHOPES practice of not counting holes smaller than ½ cm in the calculation of the net serviceability and it also suggests that any part of an irregular hole that is ½ cm wide is probably impassable and should, therefore, not be included in the determination of that hole’s ‘average width’ (see “Hole measurement” section for additional detail on the calculation of average width). When this was applied to the triangular holes tested, average widths were calculated to be 15, 21 and 42 mm for 210 mm long holes with bases of 25, 36 and 80 mm, respectively. When substituted for hole width, these average widths fit the model well as do the diameters of the circular holes tested. It appears that hole width (or average width) is an effective predictor of passability for holes of a variety of shapes; accordingly, the models described in Fig. 8 are incorporated into the net entry risk tool.

### Entry risk model

The preceding sections establish the elements of a prototype tool to predict bed net entry risk.

As presented here, the tool returns the number of *An. gambiae* females expected to enter the net per hour (plus 95% confidence limits) for a situation in which the net is under constant attack by one mosquito (i.e. a single notional mosquito outside the net which is instantly replaced by another notional mosquito outside the net when the first one enters the net). For each hole in the net, the general model is:

1 $$\#{\text{passages/}}h \, = \, \left( {\#{\text{hole encounters/}}h} \right) \, \times \, \left( {{\text{probability of hole passage/encounter}}} \right)$$

Data collected in objectives 1–3 provide key values that flesh out the general model:

#### Objective 1

The number of hole encounters per hour is related to the mean number of appearances per hour in the area around the hole. For 200 mosquitoes in the tent, average appearances per hour per 30 cm × 30 cm sampling area on the roof (FA1) = 14,450; therefore, the mean number of hourly appearances per mosquito per 30 cm × 30 cm area on the roof = 14,450/200 = 72.3.

Similarly, mean number of appearances per hour per mosquito per 30 cm × 30 cm area on the lower half of the lower third of the net (FA2—low) = 9.5, on the upper half of the lower third (FA2—high) = 1.5 and on the upper two-thirds of the net (FA3) = 0.3.

#### Objective 2

The per-appearance probability of a mosquito encountering a hole on the net side or roof is predicted by a composite value incorporating the perimeter and the area of the hole. For a given appearance of a mosquito in the 30 cm × 30 cm area the hole is in, this probability of encounter = 9 × 10^−6^ ×  (perimeter + area).

#### Objective 3

The per-encounter probability of a mosquito passing through a hole is predicted by hole width, (or diameter for round holes and average width for triangular holes). For roof holes, probability of passage for a given encounter = 0.34 ln (hole width) - 0.55 and for holes on the sides this value = 0.30 ln (hole width) - 0.52.

Incorporating these values into the general model for entry risk (1) yields four FA-specific equations predicting the hourly net entries through holes in each:

FA1: #passages/*h* = [72.3 × 9 × 10^−6^ (perimeter + area)] × [0.34ln (hole width) - 0.55]
FA2: low: #passages/*h* = [9.5 × 9 × 10^−6^ (perimeter + area)]  ×  [0.30ln (hole width) - 0.52]
FA2: high: #passages/*h* = [1.5 × 9 × 10^−6^ (perimeter + area) × [0.30ln (hole width) - 0.52]
FA3: #passages/*h* = [0.3 × 9 × 10^−6^ (perimeter + area)] × [0.30ln (hole width) - 0.52]

As a demonstration, these equations are packaged into an Excel spreadsheet (see ‘Entry risk tool’—*An. gambiae* in Additional file 1) that models the entry risk for up to five holes per FA. This tool yields a score for the net that is the average number of mosquitoes that would enter the net in an hour under the conditions discussed above. The tool also breaks the entry risk down by area of the net numerically and graphically and provides the 95% confidence limits of the entry risk that apply to each hole, to each FA and to the entire net.

When using the spreadsheet, the following should be kept in mind

- The tool presented here is based on experiments with colonized *An. gambiae* and has not been validated with wild *An. gambiae*. In addition, the tool may need to be adjusted for other vector species since they may exert different pressure patterns on the net. See, for instance, net distribution results for *Anopheles albimanus* [11].
- As previously stated, the tool calculates the hourly mean number of entries expected for a net that is under constant attack throughout the hour by one mosquito (i.e. each time the hypothetical modelled mosquito enters the net it is instantly replaced by another outside the net).
- Entry risk scores can be expressed for any time period by multiplying the values from the spreadsheet for hourly risk by the number of hours.
- Risk scores can be expressed for more than one mosquito attacking the net by multiplying the values from the tool by the number of attacking mosquitoes.
- Risk scores for any number of mosquitoes present outside the net for any number of hours can be calculated by combining the previous two measures.
- The maximum hole length or width that can meaningfully be entered in each row of the spreadsheet is 300 mm (30 cm) because all values in the model are based on sampling units of 30 cm × 30 cm. Holes longer than 300 mm can be handled by dividing them between rows in the spreadsheet. For example, a hole 400 mm long could be entered as two holes in successive lines, one with a length of, for instance, 250 mm and the other with a length of 150 mm.
- Portions of holes that span more than one FA should be apportioned accordingly and the components’ dimensions should be entered into the appropriate parts of the spreadsheet.
- Holes with a width of 5 mm or less are considered by the model to be impassable and will not affect the net score.
- Because people using bed nets in real-life are not likely to sleep just in the middle of the net or remain still and because nets often accommodate more than one person at a time, the model as presented takes a cautious approach by scoring all roof holes as though they are in FA1.
- Bed nets in normal use hang and sag in various ways not reflected by these idealized nets. This will likely affect mosquitoes as they fly along the net surfaces and interact with holes and damaged areas. Describing and accounting for these effects may lead to improvements to this tool but these are outside the scope of the present study,
- The tool assumes the net is a perfect sink for mosquitoes and does not attempt to account for mosquitoes leaving it.
- Because all experiments were done in still air which allowed the host odour plume to rise vertically (see Sutcliffe and Colborn [14]), the tool may not accurately model situations where there are significant cross drafts or air turbulence that disrupt or re-direct host odour plumes. While this may be an important consideration in some settings, the ‘still air’ condition is common; for instance, very low night time air flows (less than 0.1 m/s) have been measured in many rural houses in Africa and southeast Asia [15].
- These studies were done with untreated nets and provide a baseline for determining how much entry risk is further reduced for treated nets. A full answer to this question will require further research but a preliminary estimate is possible. Parker et al. [13] saw little or no mosquito activity 20–25 min after mosquito (susceptible *An. gambiae*) release into a tent housing a fully-charged PermaNet 2.0 net. Based on flight activity reduction alone this would translate to an hourly entry risk reduction over the untreated net prediction of 70% or more since hole encounter and hole entry are both events that require the mosquito to be active. Despite insecticide effects, it has been shown experimentally that people sleeping under holed treated nets may be bitten by insecticide-susceptible mosquitoes even though the mosquitoes are eventually killed by net contact (e.g. [16, 17]).

### Implications for bed net damage assessment

The most striking aspect of the model is the great importance it ascribes to holes on the roof of the net. This is due, in part, to the much greater mosquito pressure on the roof and, in part, to the fact that roof holes of any size are approximately 20% more passable than holes of the same size on the net sides. Surprisingly little information is available in the literature on the size and detailed distribution of damage in used nets. This makes the real-life importance of roof holes difficult to evaluate. One study [18] reported 6005 holes in 200 LLINs that had been in use for 14–20 months in Ethiopia. Of these holes, 58.7% were within 75 cm of the bottom of the net and these tended to be the largest holes. While not explicitly stated, their Fig. 6 appears to show that these nets also averaged about 2 holes/m^2^ on the roof (approximately 5 roof holes/net). Similarly, Vanden Eng et al. [19] reported that the large majority of holes in a mixed cohort of 1–3 year-old nets used in Mozambique were found on the sides with a small number (approx. 2–10% depending on hole size) appearing on the roof.

**Fig. 9:**
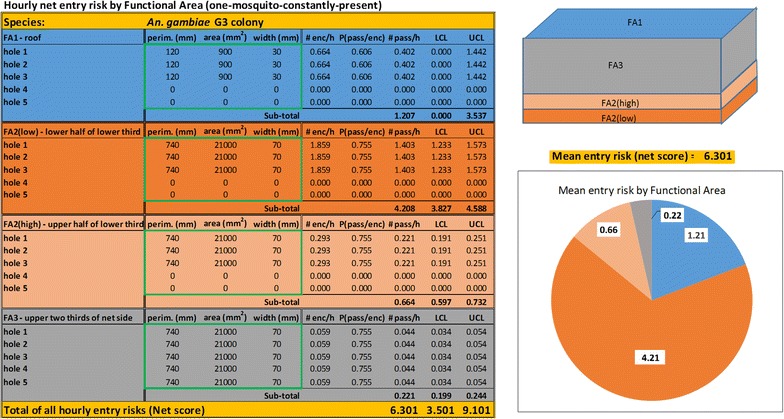
Risk assessment tool output example. Critical hole parameter values (hole perimeter, area and width in millimetres) are entered in the areas inside the *green boxes*. Other values are calculated by the spreadsheet from the algorithms described in the text. Net diagram at the *upper right* is an aid showing locations of each area colour-coded to the data entry and algorithm results display area. Below the net diagram, total entry risk is repeated from the *bottom line* of the display area. Pie chart at *lower right* diagrammatically apportions entry risk according to each Functional Area. Values entered in this example correspond to scenario described in the text

The risk assessment tool was used to score a hypothetical occupied net reflecting these damage patterns when exposed to *An. gambiae*. The modelled net has three large rectangular holes (30 cm long × 7 cm wide to reflect the long, narrow shape holes on net sides often take) in each of FA2-low and FA2-high and five such holes in FA3. It also has three smaller square holes (3 cm × 3 cm) on the roof. Assuming the external pressure on the net is one mosquito at all times, the total entry risk estimated by the model (Fig. 9) is 6.3 mosquitoes per hour of which approximately 81% is due to the holes in the sides. The remaining 19% of the risk is from the roof holes even though they only account for slightly more than 1% of the total hole area. If the roof holes were the only damage on the net, it would still admit an average of 1.2 mosquitoes per hour.

**Fig. 10:**
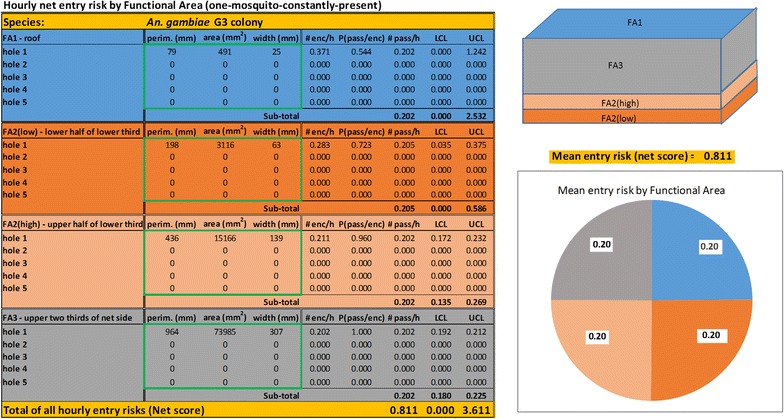
Risk assessment tool output example modelling holes representing approximately the same entry risk in each functional area of the net

**Fig. 11:**
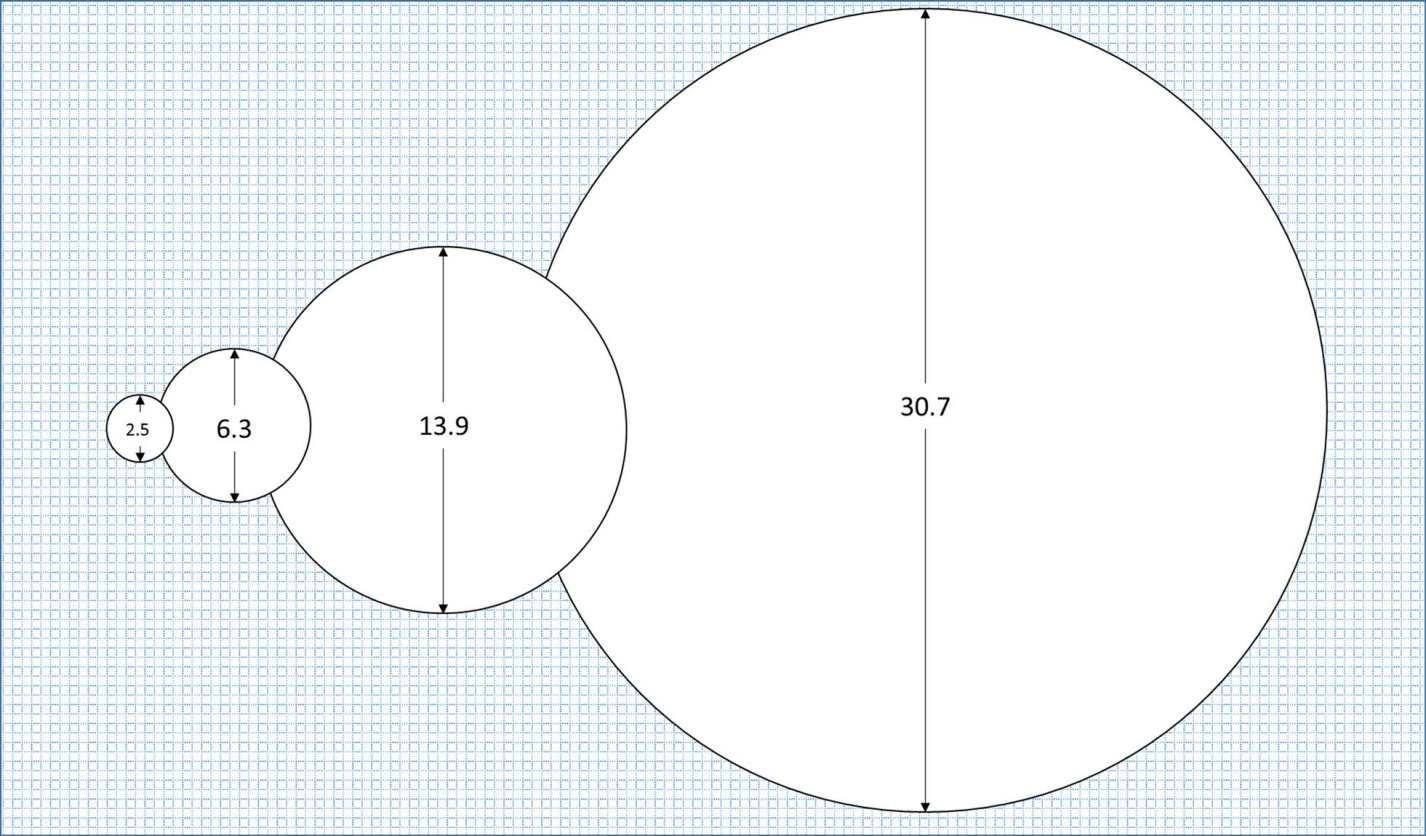
Diagrammatic representation of relative sizes (diameter in cm) of holes in (from *left to right*) FA1, FA2-low, FA2-high and FA3 representing the same entry risk according to the risk assessment tool

Various other scenarios can be modelled with the risk tool. One that is especially instructive is illustrated in Fig. 10 where values for the diameters of a single round hole in each of FA2-low, FA2-high and FA3 have been adjusted to yield holes representing the same entry risk as a 2.5 cm diameter round hole on the roof (FA1). These diameters are, respectively, 6.3, 13.9 and 30.7 cm (Fig. 11). In other words, a 2.5 cm diameter hole on the roof poses the same risk as a hole 151 times its area on the upper sides of the net. No one should (or likely could) ignore a 30 cm hole in the side of their net but this illustrates that smaller less conspicuous holes in areas of the net such as the roof are, in terms of entry risk, the same as gaping holes in other parts of the net.

The tool currently used to assess the serviceability and durability of ITNs in bed net programmes is the WHOPES-approved ‘proportionate hole index’ (pHI) [8]. The pHI method assigns each hole over ½ cm in diameter on the bed net to one of four size classes and applies a formula that multiplies the number of holes in each size class by a hole area-derived weighting factor. The resulting number is the pHI. Nets with pHIs of less than 64 are considered in ‘good’ condition and to provide “…no reduction of efficacy compared to an undamaged net” [20]. Nets with pHIs of 64–642 are classified as in ‘acceptable’ condition (reduced effectiveness but “…provides significantly more protection than no net at all…”). ‘Good’ and ‘acceptable’ nets are, in turn, classified as ‘serviceable’ in terms of protection offered while nets with pHIs greater than 642 are considered of doubtful use and to be in urgent need of replacement [20].

The following paragraphs contrast results yielded by the pHI method and by the risk assessment tool proposed here.

### Hole location

This study and others [10–13] demonstrates that various locations on the net are not under equal pressure in terms of the intensity of mosquito attack. For *An. gambiae*, the greatest pressure is on the net roof followed by the lower third of the net sides. This makes these the areas where mosquitoes are most likely to gain entry if they are damaged. Thus, it is critically important that information about the location of holes on the net is recorded and used in the net assessment. Unfortunately, the pHI field protocol discards this information after holes in the net have been counted [21]. As a result, holes on the upper sides of the net contribute to the pHI to the same degree as holes of similar sizes on the roof when, in fact, the latter are likely many times more important. Hole location information should be preserved at least in terms of the applicable FA. In turn, the mosquito distribution pattern (and, therefore, the FA breakdown) for the species of interest should be determined by experimentation since other species may not distribute around the net in the same way as *An. gambiae*. For example, sticky square collections of the sort that helped define the FA map for *An. gambiae*, showed a different configuration for *An. albimanus* which appeared to be more strongly concentrated on the net roof and did not congregate near the bottom of the net [11]. Given this, field and semi-field work to help determine FA maps for the more important malaria vector species would seem to be a high priority.

### Hole size

In the calculation of the WHOPES pHI, each hole over ½ cm is measured against a template which, whether the hole is round or not, is used to assign a diameter value to it. Hole ‘diameter’ is then used to assign each hole to one of four size classes [8]. These are: ‘smaller than a thumb’ (0.5–2 cm), ‘larger than a thumb but smaller than a fist’ (2–10 cm), ‘larger than a fist but smaller than a head’ (10–25 cm) and ‘larger than a head’ (>25 cm).

All holes in a given size class contribute equally to the calculation of the pHI even though the size classes themselves represent a large range. To test the validity of these ranges, the risk assessment tool was used to compare the *An. gambiae* entry risk represented by holes at the extremes of each of the size classes (Table 4). For the ‘smaller than a thumb’ size class, the largest hole (2 cm dia.) models as slightly more than 70 times more passable (i.e. represents 70 times more entry risk) than the smallest passable holes in the range (0.6 cm dia.). Similarly, the ‘larger than a thumb but smaller than a fist’ size class extremes represent an almost 48-fold difference in entry risk and the ‘larger than a fist but smaller than a head’ range spans a sixfold range difference in entry risk. The ‘larger than a head’ range has no upper boundary so this calculation is not strictly possible for it; however, the risk increase from the smallest value for this size class (25 cm) to the nominal mid-point of the range (30 cm), which is the value used in the pHI calculation, is 1.4×. Given the very rapid increase in hole passage rate with hole width increase, the pHI hole size classes as currently defined can probably not adequately differentiate the risk represented by holes of different sizes, especially for smaller holes. Greater resolution of hole size effect could, however, be achieved with estimates of hole width (or average width—see “Hole measurement” section).

**Table 4:** *An. gambiae* entry risks modelled for holes representing the minimum, mid-point and maximum diameters (modelled as widths) for each WHOPES pHI size class

pHI size class	Size class diameters (mm)			Modelled risk^a^			Risk range	
	Minimum	Mid-range	Maximum	Minimum	Mid-range	Maximum	Within classification	Mid-range to mid-range
Smaller than thumb	5^b^	12.5	20	0.002	0.044	0.141	70.5×	NA
Thumb-fist	20	60	100	0.141	2.074	6.711	47.6×	47.1×
Fist-head	100	175	250	6.711	20.286	41.180	6.1×	9.8×
Larger than head	250	300^c^	NA	41.180	59.177	NA	≫1.4×	2.9×

^a^Risk expressed as average number of mosquitoes expected to enter the net per hour through a round hole of given dimensions in the roof assuming the ‘one-mosquito-constantly-present’ model. Numbers expected to enter through same size holes in the sides is much smaller but ratios of risk (risk range) are similar to those for roof hole

^b^The tool assigns zero risk to holes 5 mm in diameter and smaller; therefore, minimum risk for this size class calculated using 6 mm diameter

^c^30 cm (300 mm) used as ‘middle of range’ value in pHI calculations

### Notes on hole measurement

While the four pHI hole-size classes may simplify operations in the field, the measuring method imposes circularity when, in fact, most holes in nets are not round. Vanden Eng et al. [19] examined hole shape and size in polyester and polyethylene bed nets that had been in use for up to 3 years in Mozambique. They found very few truly round holes and that treating each hole as if it was round and assigning it a diameter based on a template [8] resulted in the true areas of the predominantly elliptical holes being over-estimated by up to 400%. The present work shows that hole perimeter, actual area and width are all important predictors of the steps that result in net entry; thus, methods are needed to make these key measurements in the field in an efficient and accurate manner. The following is a non-exhaustive discussion of some of the considerations that need to be made to accomplish this.

#### Area

If holes are elliptical, or can be approximated by an ellipse, the dimensions of the major and minor axes can be used to determine area using the formula *pi* (*a* *×* *b*) where ‘a’ is half the length of the major axis and ‘b’ is half the length of the minor axis. This will work for any elliptical hole and for circular holes (for which the major and minor axis dimensions are the same) though as holes begin to approach roundness, it would be simpler and require less measurement to use the formula for area of a circle.

#### Perimeter

**Fig. 12:**
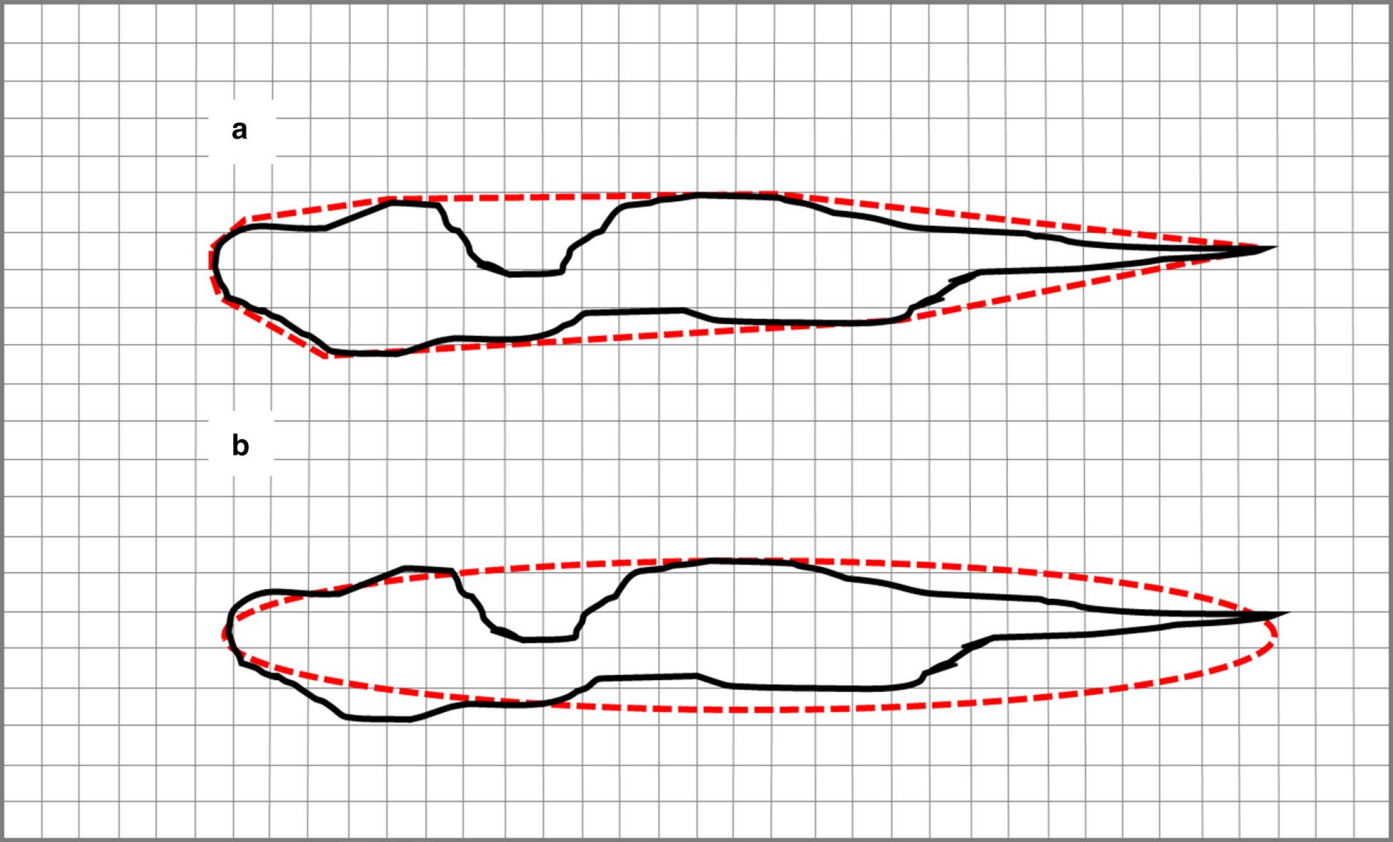
Irregularly shaped hole against a ½ cm grid template illustrating two methods of estimating hole area and effective perimeter. A. Fitted polygon (in GeoGebra)—estimated area 1378 mm^2^, actual perimeter = 293 mm, convex hull-estimated effective perimeter = 276 mm. B. Estimated dimensions from an ellipse (major axis = 135 mm, minor axis = 17 mm) superimposed on the hole—perimeter ~279 mm, area = 1802 mm^2^

The perimeter of an ellipse is not as straightforward to calculate as, for instance, the perimeter of a circle or rectangle but can be approximated to within 5% by the formula *2pi* ((*a* ^*2*^ + *b* ^*2*^ */2*)^*1/2*^) where ‘a’ is half the length of the major axis and ‘b’ is half the length of the minor axis and where the major axis is not more than three times the minor axis. Vanden Eng et al. [19] report that over 80% of elliptical holes in nets they examined fit this set of characteristics. For longer narrower ellipses, the same formula could be used but with somewhat more error. Where the major axis is very long in comparison to the minor axis (i.e. for very narrow holes), the perimeter might be better estimated by simply doubling the length. Again, as the hole widens to roundness, the formula for the perimeter of the ellipse will still work but it would then be simpler to treat it as a circle. Holes with more complex shapes would present a special case since their true perimeters could be large and lead to unrealistic estimates of encounter. In these cases, the effective perimeter could be estimated by calculating the perimeter of the ‘convex hull’ [22] that bounds the hole or by fitting an ellipse to the hole profile (Fig. 12).

#### Width

**Fig. 13:**
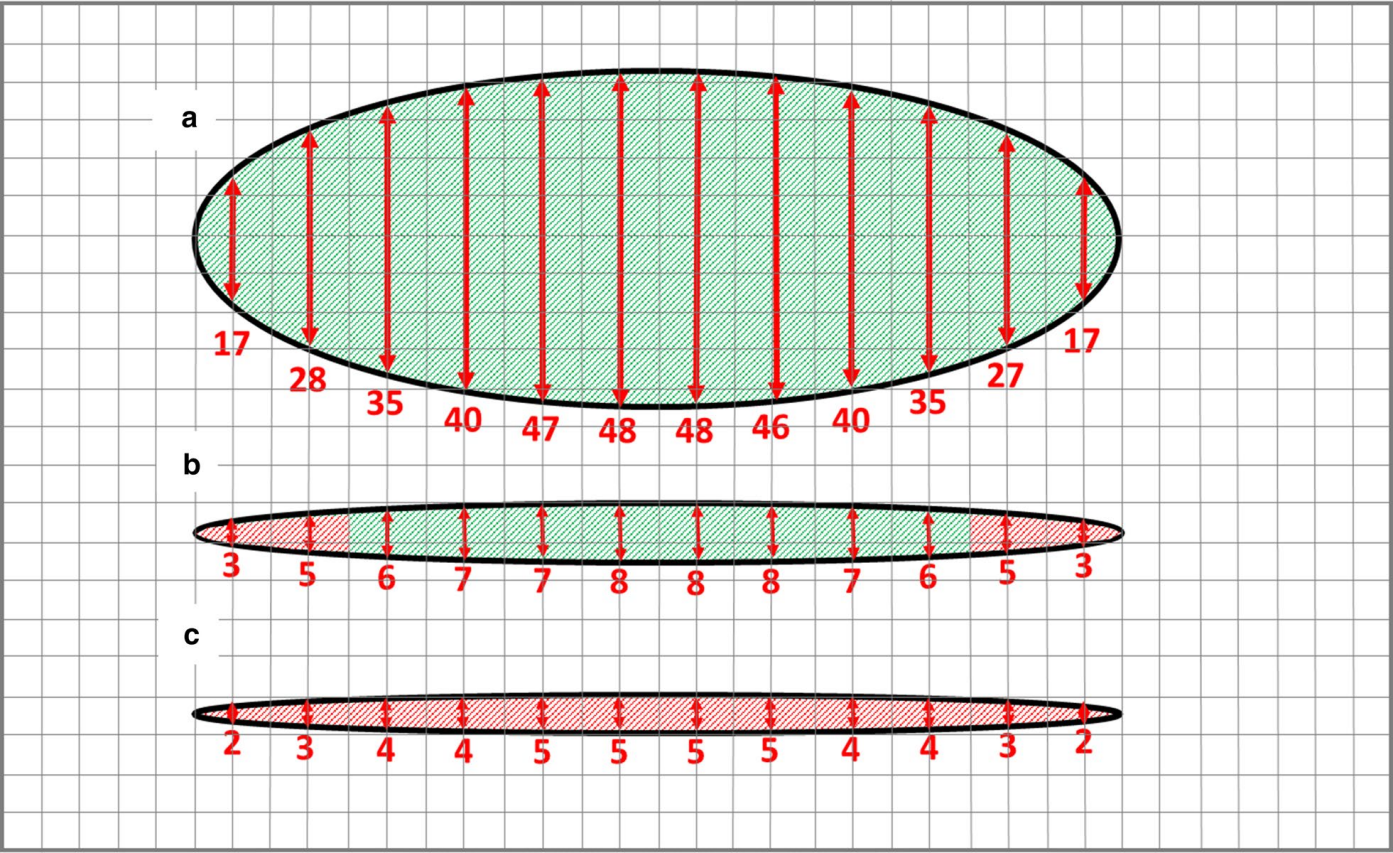
Three ellipses representing types of holes on a bed net each superimposed on a ½ cm grid for purposes of hole measurement. Widths of mid-point of each 1 cm section indicated in mm. **a** A large open ellipse with major axis = 120 mm and minor axis = 50 mm. Perimeter ~289 mm; area = 4710 mm^2^. Hole width is greater than ½ cm at all points along its length meaning that the risk assessment tool considers all parts of the hole passable (*green crosshatch*) for *An. gambiae*. Average hole width = sum of mid-point widths for all 1 cm sections/# of sections >½ cm wide = 36 mm. **b** Longer narrower elliptical hole with major axis = 120 mm and minor axis = 8 mm. Perimeter ~267 mm; area = 754 mm^2^. Two 1 cm sections at each end (*red crosshatch*) have mid-point widths less than or equal to ½ cm and are considered impassable by the tool. Average hole width = 7 mm. C. Very narrow elliptical hole with major axis = 120 mm and minor axis = 5 cm. Perimeter ~267 mm (by formula) or 240 mm by doubling length (see text); area = 471 mm^2^. None of the hole’s 1 cm sections has a mid-point width >½ cm meaning that the tool considers the entire hole impassable (*red crosshatch*); average width = 0 mm. NOTE: In cases where a hole, irrespective of actual shape, does not have a section width dimension >½ cm, time need not be wasted measuring its width

**Fig. 14:**
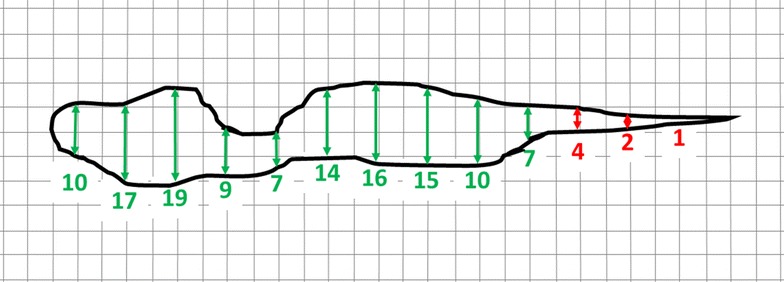
Irregular hole superimposed on ½ cm template to determine average width. All 1 cm sections except the rightmost three are greater than ½ cm wide. Average width = 12 mm

A hole, or any given part of a hole, must be over ½ cm wide for the tool to consider it passable; therefore, holes (or parts of holes) narrower than this need not be entered in the model (although it may still be advisable to record their existence and location since they reflect overall net condition since, in time, small holes will become larger). In the case of a circular hole, diameter serves adequately as its width but for elliptical or irregular holes, an average width is required. This means measuring hole width at intervals along its length and taking the average of all section widths wider than ½ cm. The more frequent the intervals, the more accurate the estimate will be but the more labour intensive the task will become. In the examples (Figs. 12, 13, 14), measurements are taken every centimetre.

#### Hole measurement methods

In the absence of technological aids, hole dimensions would have to be estimated manually but since most net holes have fairly simple shapes this should be feasible. Measurements could be done in a manner similar to how hole size is currently determined for the WHOPES pHI. That is, with reference to a template held behind the hole as a guide [21]. In this case the template would be of a 1 or ½ cm squared grid and, perhaps, a selection of hole shapes and sizes if there was a typical range. In practice, some labour savings may be possible in this task since holes over a certain width (approx. 70 mm on the roof and 110 mm on the sides), could all be considered effectively 90 or 100% passable without having to measure them all.

There are also several technological aids available that may reduce the labour intensiveness and increase the accuracy of this process. One such approach is with image analysis of digital photographs of the holes. Several suitable image analysis packages are available in which it would be possible to develop macros or plugins for measuring hole dimensions such as those discussed here. For example, ImageJ is freely available for download from the National Institutes of Health [23]. A key part of this approach would be a standardized method of taking photographs of net damage that would yield images of sufficient quality that include a scale for calibrating measurements. Smart pens or measuring apps for smart phones or hand held tablets are also available that might be applied to this purpose.

There are also a number of graphing utilities that could be useful in this context. One such utility (available online or downloadable to use offline in the Chrome web browser) which allows the user to model ellipses of virtually any size, is available on the ‘Math Open Reference’ website [24]. Another is the (freely downloadable for non-commercial use) ‘GeoGebra’ graphing calculator [25] with which virtually any shape can be modelled and its dimensions (area, perimeter, width) measured. Digital photographs (e.g. of net damage) can also be opened in GeoGebra which could then be used make the key measurements.

### Net serviceability versus durability

This tool provides a way of estimating the rate of mosquito entry into a damaged net. Depending on how damage is distributed, the tool shows that a net with relatively little damage (e.g. a few small holes on the roof) may have a higher entry risk than a net with much more extensive damage (e.g. several medium-sized or large holes on the upper sides). Thus, the tool should be an effective way of determining and comparing net protective value (net serviceability) but it would not necessarily reflect the overall physical condition (durability) of bed nets. The distinction between net serviceability and durability is important and each quality must be measured and applied appropriately. Clearly, the entry risk tool is appropriate for questions relating to net serviceability and the timing of net replacement once the nets are in place. On the other hand, the WHOPES pHI or the composite index [19] may be better suited to measuring overall durability. Durability information, in turn, should be used to inform procurement decisions since, other things being equal, programmes should want to spend their money on the bed nets that appear to be most likely to survive the wear and tear of daily use.

## Conclusions

In its report to the Malaria Policy Advisory Committee (MPAC) on issues relating to estimates of bed net survival in the field, the Vector Control Technical Expert Group (VCTEG) [26] lists several outstanding needs. One of these is “*To understand better the determinants of mosquito entry into a damaged net and to improve*—*if needed*—*the weighting system for hole counts in the proportionate hole index, there is a need to study the relationship between hole size and position on an effective LLIN and the influence of total net size compared to the size of the hole*.” The prototype tool presented here is the first of its kind based on direct observations and measurements of mosquito behaviour around bed nets and is a direct response to this stated need. While it may be possible to refine its accuracy for different mosquito species and specific circumstances, the tool even as presented here should provide more meaningful estimates of net protectiveness than anything else currently available. The estimates the tool provides may be used in various ways. Most simply, they could form the basis of a decision tree scheme similar to the way the pHI is currently used to establish thresholds of net ‘serviceability’. This would set ‘acceptable’ levels of mosquito entry into the net. While this would be expressed in meaningful terms (mosquito entry as opposed to simple damage), it would suffer from some of the same drawbacks as the pHI thresholds; namely, the thresholds would be arbitrary. In addition, this would only measure the risk of mosquitoes entering the net, not the risk of malaria per se. The tool’s ‘value added’ is that it provides specific estimates of the numbers of mosquitoes that will enter damaged nets making it a quantitative predictive tool. Accordingly, it may be possible to use the tool in combination with entomological and epidemiological data (such as local mosquito densities, sporozoite rates, etc.) to determine actual locale/epidemiological situation-specific malaria risk levels posed by worn nets. When used in this way, the amount and type of damage that defines a ‘failed net’ could be expressed in terms of malaria risk meaning that it is likely that a net that is judged as ‘failed’ in one epidemiological setting (e.g. where sporozoites rates are high) might be judged to be serviceable in another (e.g. where sporozoites rates are low). This would allow a nuanced approach to bed net assessment and replacement that could help maximize public health benefit while minimizing net replacement costs.

## Additional file

**Additional file 1** Working version of the entry risk tool in Excel format. See Figure 9 for instructions in its use and interpretation.

## Abbreviations

FA: functional area (of bed net)
HEPA: high efficiency particulate air
ITN: insecticide-treated bed net
LLIN: long lasting insecticide-treated net
MPAC: Malaria Policy Advisory Committee
VCTEG: Vector Control Technical Evaluation Group
pHI: proportionate hole index
WHO: World Health Organization
WHOPES: World Health Organization Pesticide Evaluation Scheme
